# Applications and Limitations of Equilibrium Density Gradient Analytical Ultracentrifugation for the Quantitative Characterization of Adeno-Associated Virus Vectors

**DOI:** 10.1021/acs.analchem.3c01955

**Published:** 2024-01-02

**Authors:** Kiichi Hirohata, Yuki Yamaguchi, Takahiro Maruno, Risa Shibuya, Tetsuo Torisu, Takayuki Onishi, Hideto Chono, Junichi Mineno, Yuan Yuzhe, Kiyoko Higashiyama, Kyoko Masumi-Koizumi, Kazuhisa Uchida, Takenori Yamamoto, Eriko Uchida, Takashi Okada, Susumu Uchiyama

**Affiliations:** † Department of Biotechnology, Graduate School of Engineering, Osaka University, 2-1 Yamadaoka, Suita, Osaka 565-0871, Japan; ‡ Takara Bio Inc., 7-4-38 Nojihigashi, Kusatsu, Shiga 525-0058, Japan; § Graduate School of Science, Technology and Innovation, Kobe University, 1-7-49 Minatojima Minamimachi, Chuo-ku, Kobe 650-0047, Japan; ∥ Division of Molecular Target and Gene Therapy Products, National Institute of Health Sciences, 3-25-26 Tonomachi, Kawasaki-ku, Kawasaki-city, Kanagawa 210-9501, Japan; ⊥ Institute of Medical Science, The University of Tokyo, 4-6-1, Shirokanedai, Minato-ku, Tokyo 108-0071, Japan

## Abstract

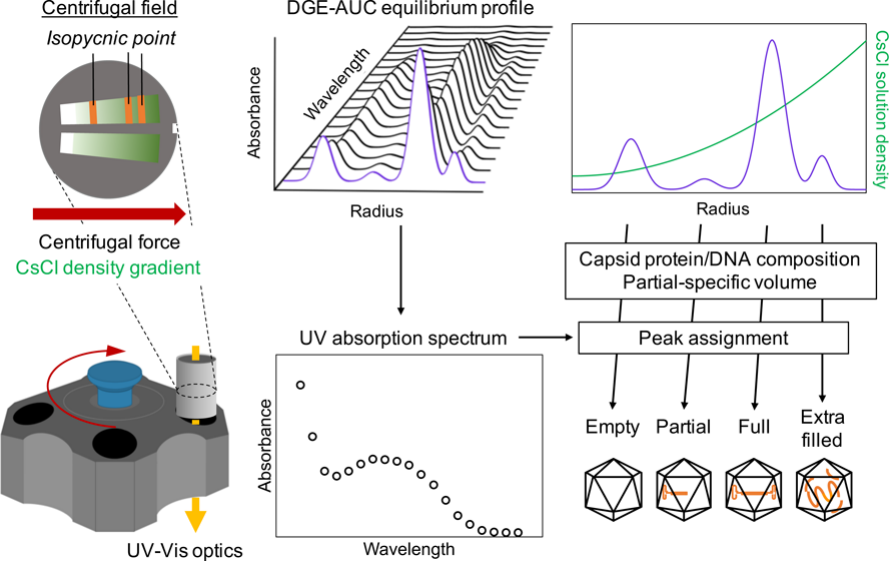

Adeno-associated virus (AAV) vectors are produced as a mixture of the desired particle (full particle, FP), which is filled with the designed DNA, product-related impurities such as particle without DNA (empty particle, EP), and aggregates. Cesium chloride or iodixanol equilibrium density gradient ultracentrifugation (DGE-UC) has been used for the purification of AAV vectors. DGE-UC can separate FP from impurities based on the difference in their buoyant densities. Here, we report the applications and limitations of equilibrium density gradient analytical ultracentrifugation (DGE-AUC) using a modern AUC instrument that employs DGE-UC principles for the characterization and quantitation of AAV vectors. We evaluated the quantitative ability of DGE-AUC in comparison with sedimentation velocity AUC (SV-AUC) or band sedimentation AUC (BS-AUC) using AAVs with different DNA lengths and different serotypes. DGE-AUC enabled the accurate quantification of the ratio of FP to EP when the AAV vector primarily contains these particles. Furthermore, we developed a new workflow to identify the components of separated peaks in addition to FP and EP. Ultraviolet absorption spectra obtained by multiwavelength detection can also support peak assignment following component identification. DGE-AUC experiments for AAV vectors have limitations with regard to minor components with low absorption at the detected wavelength or those with a density similar to that of major components of AAV vectors. DGE-AUC is the only analytical method that can evaluate particle density heterogeneity; therefore, SV-AUC or BS-AUC and DGE-AUC are complementary methods for reliable assessment of the purity of AAV vectors.

## Introduction

Adeno-associated virus (AAV) vectors are the leading platform for the delivery of *in vivo* gene therapies.^1^ AAV comprises three viral proteins (VPs), VP1, VP2, and VP3, which form a 60-mer icosahedral capsid structure, in which approximately up to 4.7 kb of DNA can be encapsidated. AAV has the advantages of nonpathogenicity in humans, long-term gene expression in nondividing cells, and transduction into various tissues by different serotypes. In the upstream process of AAV vector production, AAV vectors are generated as a mixture of the desired particle (full particle, FP) encapsidates single-strand or self-complementary DNA (ssDNA or scDNA), particle without DNA (empty particle, EP) or with some DNA fragments (partial particle, PP), particle with longer fragments or higher numbers of DNA (extrafilled particle, ExP), and aggregates. Despite extensive development in this field, complete removal of impurities other than the desired FP remains difficult in the downstream process; thus, AAV vector-based products potentially contain some impurities even after purification. The presence of EP can reduce transduction efficiency and increase the risk of immunogenicity,^2^ and the impact of impurities on biological activities and adverse effects is still not fully understood. In fact, clinical applications can be hindered as a result of quality-related issues with AAV vectors.^3^ Guidance from the Food and Drug Administration (FDA) states that the physicochemical properties of drug products (e.g., the aggregation state and the ratio of infectious to noninfectious particles or full to empty particles) should be confirmed.^4^ It is thus essential to accurately assess the purity and size distribution of the AAV vector drug products. Furthermore, the manufacturing process could be improved by monitoring changes in the size distribution throughout the AAV vector production. To date, various methods have been reported to identify and quantify FP and EP.^5^ In addition to conventional approaches, such as enzyme-linked immunosorbent assay and quantitative polymerase chain reaction, chromatography-based techniques and negative stain transmission electron microscopy have been employed. Cryo-electron microscopy, mass photometry, and charge detection mass spectrometry have also recently emerged as alternative methodologies.^6−8^ Compared with these orthogonal methods, two analytical ultracentrifugation (AUC) methods, sedimentation velocity analytical ultracentrifugation (SV-AUC) and band sedimentation analytical ultracentrifugation (BS-AUC), have been established by Maruno et al. to enable the quantification not only of FP and EP but also of other components with high accuracy, reproducibility, and serotype-independence.^9,10^

Another AUC method, equilibrium density gradient analytical ultracentrifugation (DGE-AUC), might be applicable to AAV vector characterization. The principle of DGE-AUC is based on equilibrium density gradient ultracentrifugation (DGE-UC) while utilizing the powerful functions of a modern AUC instrument equipped with a recently developed optical system. Under the centrifugal field, compounds such as cesium chloride (CsCl) and iodixanol generate a concentration gradient, leading to a density gradient formation in the direction of the centrifugal force. The macromolecule in the solution migrates depending on the difference between its buoyant density and solution density. The macromolecule finally forms a band at the isopycnic point, where its buoyant density is equal to the solution density at equilibrium. A study by Meselson and Stahl in 1958, one of the most elegant experiments in biology, first performed CsCl-DGE-AUC in Model E analytical ultracentrifuge.^11^ They provided firm evidence that DNA replication is a semiconservative mechanism by measuring slight density differences in DNA. Since then, DGE-UC has been extensively utilized for the isolation of biological substances, for example, viruses composed of proteins and nucleic acids, because it offers highly resolved separation based on buoyant density. In fact, DGE-UC has been applied to separate FP from impurities during AAV vector purification, while examples of DGE-AUC application have been reported for adenovirus preparation characterization using previous AUC instruments.^12−14^ By employing a modern AUC instrument with a multiwavelength (MW) detection system that offers increased sensitivity, it is possible to derive ultraviolet (UV) absorption spectra for the observed peaks to clarify the components in AAV vectors. DGE-AUC also confers the advantage that the obtained data can be analyzed by commercial software such as Microsoft Excel and Origin or even with an in-house Python program, which is important for the quality control of viral vectors under current Good Manufacturing Practice (cGMP)-compliant conditions. Despite DGE-AUC being a promising analytical method, component identification and quantification procedures remain to be established.

Here, we evaluated the advantages and limitations of applying DGE-AUC with a MW detection (MW-DGE-AUC) to AAV vector characterization. First, we examined the experimental conditions of MW-DGE-AUC for AAV analysis. Then, we confirmed the validity of the peak assignments by characterizing the observed peaks in MW-DGE-AUC and also assessed the quantitation ability of DGE-AUC by determining the ratio of FP to EP (F/E ratio). Using AAV vectors with different serotypes and ssDNA lengths, which had been well-characterized by SV-AUC and BS-AUC, we developed a component identification workflow by MW-DGE-AUC. Finally, we proposed an optimization method for the MW-DGE-AUC experimental conditions for AAV vector analysis as well as the optimum purification conditions by CsCl-DGE-UC.

## Experimental Section

### Materials

Laboratory-grade AAV vectors (serotypes 2, 6, and 8) and EP of AAV2 (AAV2-EP) in phosphate-buffered saline (PBS) were purchased from Vector Builder (Chicago, IL) and Addgene (Watertown, MA). AAV5 vector was supplied by Manufacturing Technology Association of Biologics (Tokyo, Japan). PBS (Thermo Fisher Scientific, Waltham, MA), poloxamer-188 (BASF Japan, Tokyo, Japan), sodium chloride (FUJIFILM Wako Pure Chemical, Osaka, Japan), water isotopically enriched to >98 atom % H_2_^18^O (Rotem, Arava, Israel), and cesium chloride (Nacalai Tesque, Kyoto, Japan) were used. When iodixanol remained in the AAV vector stock solution, the buffer was exchanged with PBS containing 0.001% poloxamer-188 by centrifugation using an Amicon Ultra-0.5 centrifugal filter unit, 100 kDa MWCO (Merck Millipore, Burlington, MA) before the AUC experiments. For the characterizations of FP, AAV8EGFP was manufactured and completely purified as described in the Supporting Information.

### UV Measurement

UV measurements were performed using a UV-1900 (Shimadzu, Kyoto, Japan) for the AUC experiments, and data were collected at 210–350 nm every 1 nm using a 1 cm light path cuvette.

### DGE-AUC

AAV vectors were dissolved in CsCl/PBS solution with 0.001% poloxamer-188 to a final absorbance at a 1 cm path length of approximately 0.1 at 230 nm. The final CsCl concentration in the solution was adjusted to 2.75 M. A volume of 390 μL of AAV solution was loaded into the sample sector equipped with sapphire windows and a 12 mm double-sector charcoal-filled epon centerpiece (Beckman Coulter, Brea, CA). Moreover, a volume of 400 μL corresponding to 2.75 M CsCl/PBS solvent with 0.001% poloxamer-188 was loaded into the reference sector. Data were collected at 20 °C using Optima AUC (Beckman Coulter) at 42,000 rpm with a UV detection system. The wavelength for UV detection was set within the range of 230–320 nm every 5 and 350 nm for MW-DGE-AUC. Data were collected every hour with a radial increment of 10 μm. The collected DGE-AUC data at equilibrium were analyzed by using the OriginPro 2023 (10.0) Software (Origin Lab, Northampton, MA). For the imported data set, a linear baseline was created and subtracted by selecting “Subtract Baseline” as the Goal with the Peak Analyzer. Then, for the baseline subtracted data set, peaks were fitted and peak areas were calculated using the “Multiple Peak Fit” tool by selecting the peak center location and setting the peak function as Gaussian. The root-mean-square deviation (RMSD) between the DGE-AUC profiles obtained at different times was calculated to assess the time required for the CsCl density gradient to reach equilibrium. For AAV vectors with different serotypes and ssDNA lengths, the RMSDs between the profiles every 2 h up to 24 h are shown in Figure S1. The RMSD was close to zero at approximately 12 h, indicating that the migration of AAV particles associated with the formation of the CsCl density gradient reached equilibrium in 12 h. Thus, the DGE-AUC equilibrium profiles were obtained 12 h after the beginning of centrifugation for all AAV vectors in this study. Averaging of scans was considered through repeated scanning after reaching equilibrium. As shown in Figure S2a, the single scan data provided result sufficient for the reliable quantitation, as evident from the signal-to-noise ratio value. Also, the triplicate experiments showed high reproducibility for the peak areas of the observed peaks (Figure S2b). The population of each peak was corrected by considering sector-shaped centerpiece geometry as provided in Figure S3.

### SV-AUC and BS-AUC

SV-AUC and BS-AUC experiments and analyses were performed according to previously published methods.^9,10^ Detailed experimental procedures are provided in the Supporting Information. Data analyses were carried out using the program SEDFIT.^15^ Figures of *c*(*s*) distributions were generated using the program GUSSI.^16^

### Molecular Weight and Partial-Specific Volume Calculation

The theoretical molecular weight (Mw) and partial-specific volume (*vbar*) in water of AAV-EP were determined from the amino acid compositions of each VP (the VP1/VP2/VP3 ratio was considered to be 5:5:50) using the program SEDNTERP.^17^ The theoretical Mw of ssDNA was calculated from the DNA compositions. Furthermore, the Svedberg equation was used to calculate the Mw from the experimentally determined *s*-value in the peak assignment of AAV8 vectors by MW-BS-AUC
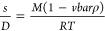
1where *s* is the sedimentation coefficient (*s*-value), *D* is the diffusion coefficient, *M* is the Mw, *vbar* is the particle partial-specific volume, ρ is the solvent density, *R* is the gas constant, and *T* is the absolute temperature.

The *vbar* of AAV-FP in water can be theoretically calculated using the following equation
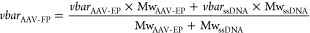
2where *vbar*_AAV-FP_, *vbar*_AAV–EP_, and *vbar*_ssDNA_ are the partial-specific volume of AAV-FP, AAV-EP, and ssDNA, respectively. 0.52 cm^3^/g was used as *vbar*_ssDNA_ based on a previous study.^9^ Mw_AAV–FP_, Mw_AAV–EP_, and Mw_ssDNA_ are the Mw of AAV-FP, AAV-EP, and ssDNA, respectively.

## Results and Discussion

### Characterization of the Components Observed in the DGE-AUC Equilibrium Profile

**Figure 1:**
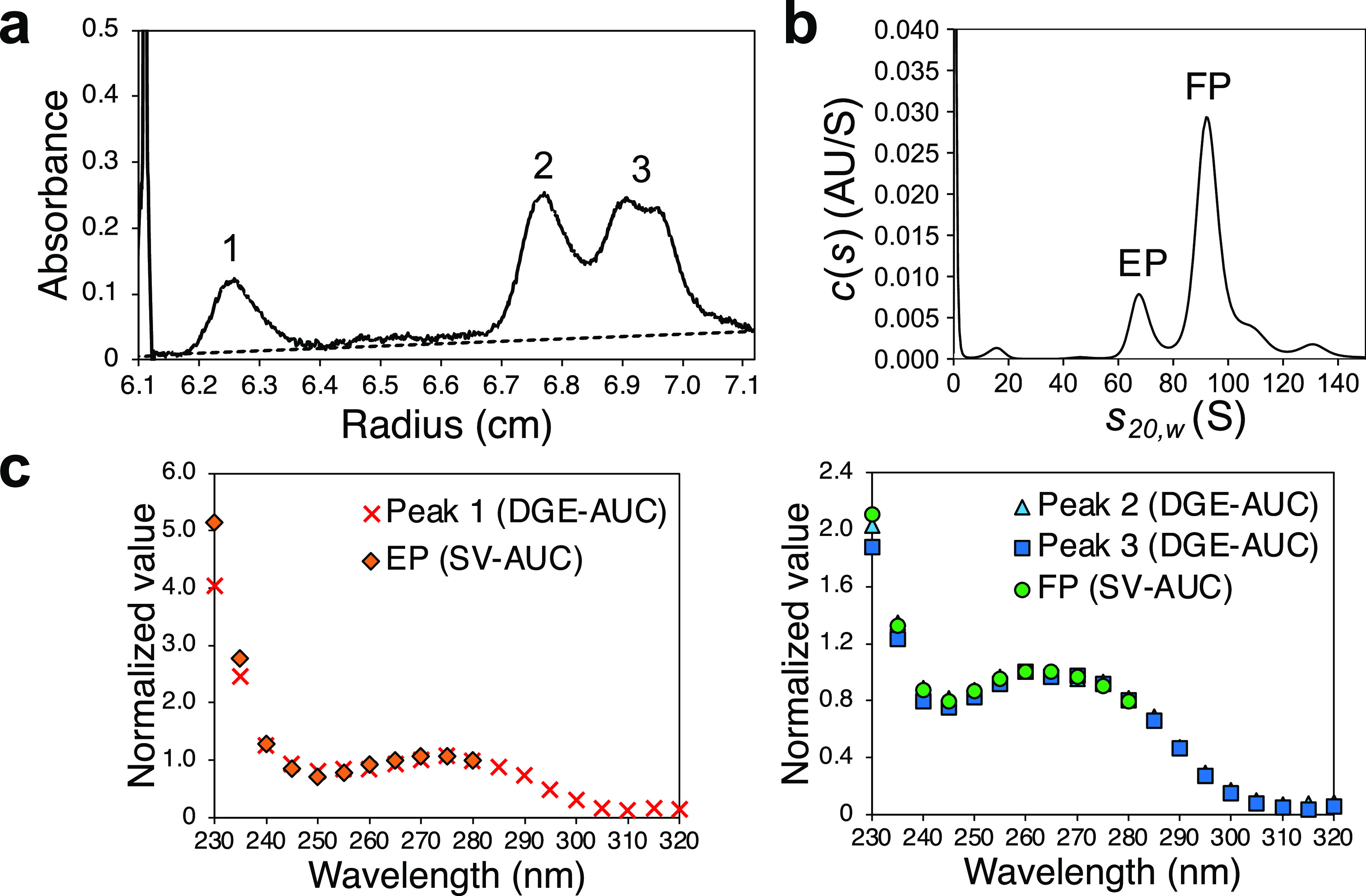
Assignment of the peaks observed in the DGE-AUC equilibrium profile. (a) DGE-AUC equilibrium profile for AAV2EGFP detected at 230 nm. The radius represents the distance from the center of the rotation. The large peak observed at around 6.1 cm indicates the meniscus position of the solution. The dashed line represents the baseline for subtraction. (b) *c*(*s*) distribution of AAV2EGFP obtained by SV-AUC detected at 230 nm. (c) Overlaid plots of the peak areas for EP and the maximum peak values for peak 1 at each wavelength normalized at 280 nm (left) and the overlaid plots of the peak areas for FP and the maximum peak values for peaks 2 and 3 at each wavelength normalized at 260 nm (right).

We established MW-DGE-AUC for AAV characterization using the methods described in the Experimental Section and performed MW-DGE-AUC using AAV2EGFP. In the DGE-AUC equilibrium profile (Figure 1a), three peaks, peaks 1–3, were observed in order from the axis of the rotation. On the other hand, *c*(*s*) distribution obtained by SV-AUC showed two major peaks at 68.5 and 95.0 S (Figure 1b). As AAV2-EP and AAV2EGFP have slightly larger molecular weights than the previously reported AAV5-EP (67.3 S) and AAV5ZsGreen1 (93.7 S), respectively,^9^ the 68.5 and 95.0 S peaks corresponded to EP and FP, respectively. With MW-SV-AUC, the normalized peak areas at each wavelength for EP and FP are plotted in Figure 1c. The DGE-AUC equilibrium profile showed a gradual increase in the baseline as the radius from the axis of the rotation increased. This phenomenon could be attributed to the presence of impurities that are low-molecular-weight species (LMWS), such as VP and/or DNA fragments, as they form a concentration gradient similar to that of CsCl. Since the baseline increase impaired the accurate estimation of the peak area and A260/A280 value, we subtracted the baseline during data analysis in this study, as shown in Figure 1a. With MW-DGE-AUC, the normalized maximum peak values for peaks 1–3 at each wavelength were plotted, and the overlaid plotting results of peak 1 and EP were matched with each other, and the overlaid plotting results of peaks 2 and 3 were matched with that of FP (Figure 1c). Consequently, peaks 2 and 3 were assigned as FP, while peak 1 was assigned as EP.

It is challenging to unambiguously map each minor peak at ∼110 and 132.5 S in *c*(*s*) distribution obtained by SV-AUC to each peak in the DGE-AUC equilibrium profile. Although conclusive assignment was difficult even with A260/A280 information due to the weak signal intensity, we interpreted it as follows. The ∼110 S component has two possibilities: ExP, which should contain about 4800 bases of DNA based on a linear correlation between the *s*-value and the encapsidated DNA length,^18^ and place at ∼7.07 cm; and/or the FP-EP dimer, which should place at ∼6.55 cm. The expected encapsidated DNA length of the 132.5 S peak is about 8200 bases, which is over the limitation of encapsidation into an AAV particle. In general, the *s*-value ratio of the dimer to monomer is 1.45,^19^ so that the 132.5 S component corresponds to the FP-FP dimer, which overlapped with peak 2 or 3.

**Figure 2:**
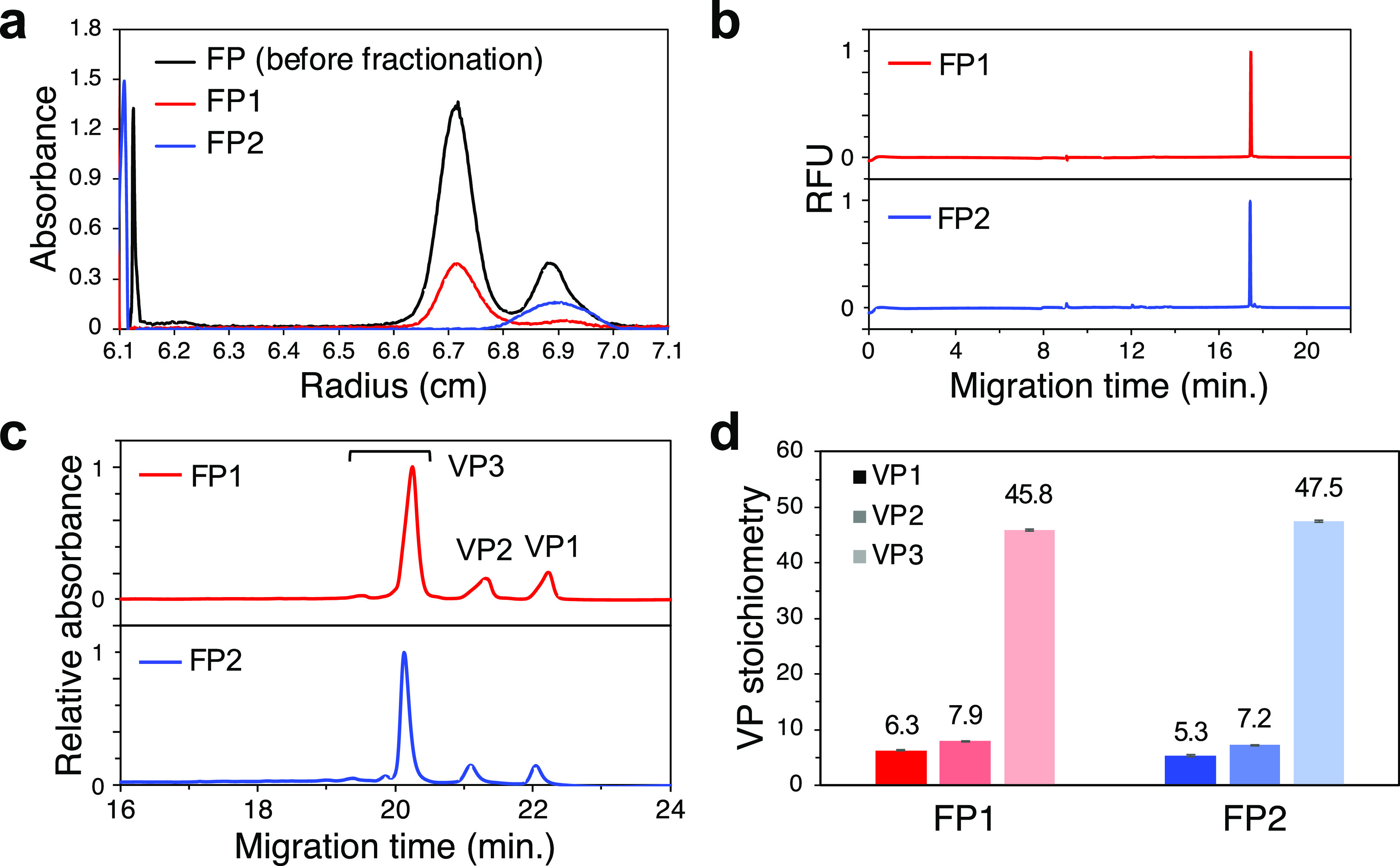
Characterizations of FP1 and FP2. (a) DGE-AUC equilibrium profiles for FP before fractionation, FP1, and FP2 of AAV8EGFP detected at 230 nm. (b) CGE electropherograms of encapsidated ssDNA of FP1 and FP2. (c) CGE electropherograms of FP1 and FP2 for VP components detected at 214 nm normalized with respect to the VP3 peak top value. (d) VP stoichiometries of FP1 and FP2 determined from CGE measurements for VP components. The detailed procedures of CGE measurements and analyses are provided in the Supporting Information.

The DGE-AUC equilibrium profiles of AAV5ZsGreen1 and AAV8EGFP also showed that the existence of two major peaks corresponding to FP was confirmed to be the same as AAV2EGFP (Figures S4 and 2a). Buoyant density heterogeneity of FP has been observed in the *Parvoviridae* family, including wild-type AAV; however, its origin was unclear.^20−23^ Another previous study proposed the different buoyant densities of two AAV8-FP encapsidating scDNA caused by different VP stoichiometries.^24^ A recent study showed that the VP ratio varies depending on as yet uncharacterized mechanisms.^25^ To this end, we carried out two-cycle CsCl-DGE-UC to completely separate and fractionate FP of AAV8EGFP into FP1 (low buoyant density) and FP2 (high buoyant density) (Figure S5). The DGE-AUC equilibrium profiles of both FP1 and FP2 showed one major peak, corresponding to low and high buoyant density FPs, respectively (Figure 2a). The encapsidated DNA length was the same between FP1 and FP2 by capillary gel electrophoresis (CGE) measurement as shown in Figure 2b, while the determined VP ratio from the peak area in the CGE electropherogram for VP components was VP1/VP2/VP3 = 6.3:7.9:45.8 for FP1 and VP1/VP2/VP3 = 5.3:7.2:47.5 for FP2 (Figure 2c,2d). Therefore, we concluded that the cause of the different buoyant density of FP1 and FP2 is the difference in VP stoichiometry. Recently, our study on AAV2 supports the present conclusion.^26^ Thus, DGE-AUC is currently a powerful analytical method that can evaluate FP with different VP stoichiometries for AAV vector characterization.

### Quantification of the F/E Ratio by DGE-AUC

**Figure 3:**
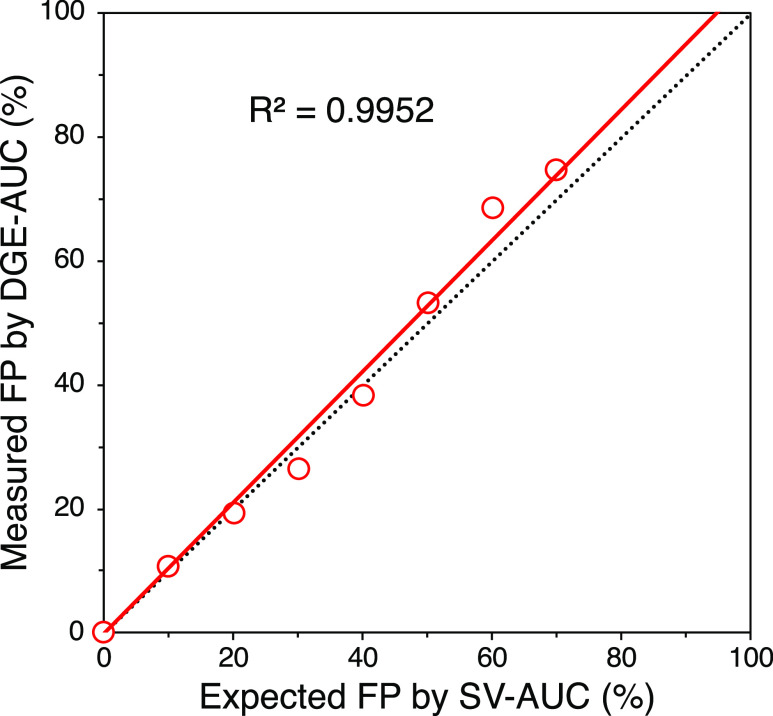
Linearity of F/E ratio quantification by DGE-AUC. Red circle plots represent the FP amount measured by DGE-AUC for each sample, and the red solid line represents the regression line of the plots. The F/E ratio was calculated under conditions whereby the total of EP and FP equaled 100%. The black dotted line indicates the *y* = *x* line.

Since the F/E ratio is regarded as one of the critical quality attributes (CQAs) of AAV vectors, we examined the linearity of the F/E ratio quantitation by DGE-AUC using the samples prepared by mixing AAV2-EP and AAV2EGFP at different ratios. The F/E ratios of these samples were quantified by DGE-AUC and then compared with the expected F/E ratios from the SV-AUC results. The amount of FP determined by DGE-AUC was the sum of the amount of FP with different VP stoichiometries. Figure 3 shows the linear correlation of the percentages of the F/E ratio between DGE-AUC and SV-AUC. These results indicate that DGE-AUC is a quantitative analytical method for F/E ratio determination equivalent to SV-AUC.

### Peak Assignments of AAV8 Vectors by MW-BS-AUC

**Figure 4:**
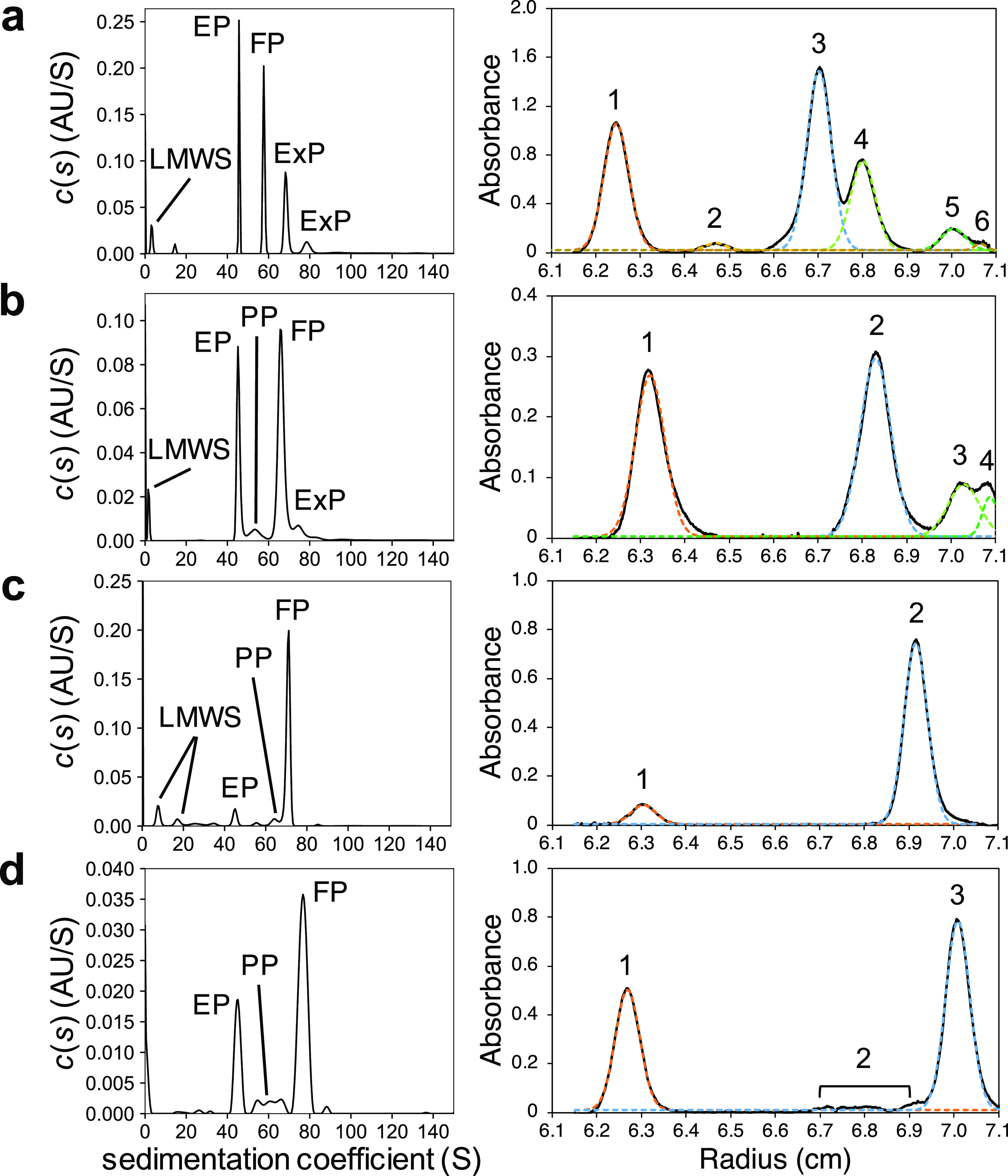
Comparison of *c*(*s*) distributions by BS-AUC and the DGE-AUC equilibrium profiles. *c*(*s*) distributions (left) and the DGE-AUC equilibrium profiles (right) of (a) AAV8H4C1, (b) AAV8FIXp, (c) AAV8oScarlet, and (d) AAV8mCherry-EGFP. All results were detected at 230 nm. The DGE-AUC equilibrium profiles were Gaussian peak fit results after baseline subtraction. The black solid line represents the profile after baseline subtraction. The colored dashed lines represent the fitted peaks. The DGE-AUC equilibrium profiles before the baseline subtraction are shown in Figure S9.

The characterization of four AAV8 vectors was performed by MW-BS-AUC and compared with the component identification results from MW-DGE-AUC presented in the following sections. *c*(*s*) distributions and peak assignment results are summarized in Figure 4 (left) and Table 1. The Mws of peaks at 45 S for all AAV8 vectors were calculated by using eq 1. Compared with the theoretical Mw of AAV8-EP, these peaks were assigned as EP. The Mws were calculated for the main peaks except those of EP, for all AAV8 vectors in the same manner, and the peaks were confirmed as FP by comparison with the theoretical Mws of FP. Plotting the *s*-value against the encapsidated full-length DNA led to a linear correlation (Figure S6). Peaks with *s*-values between EP and FP or greater than FP that fit this linear relationship were assigned as PP and ExP, respectively, also taking the A260/A280 value into consideration. It is worth noting that the component with the largest *s*-value for each AAV8 vector had inaccurate A260/A280 values by MW-BS-AUC.^10^ Peaks with *s*-values smaller than that of EP were assigned as LMWS.

**Table 1:** Peak Assignments of AAV8 Vectors by MW-BS-AUC

AAV8H4C1 (1487 bases)	Component	LMWS	EP	FP	ExP	ExP
	*s*_w_ (S)	3.3	45.8	57.7	68.5	78.9
	Mw (kDa)		3684.2 (3735.3)	4363.6 (4195.5)		
	A260/A280	0.92	0.58	1.10	1.28	1.29
	peak area (%)	5.4	26.1	34.0	26.9	7.7
AAV8FIXp (2712 bases)	component	LMWS	EP	PP	FP	ExP
	*s*_w_ (S)	1.8	45.5	53.8	66.2	75.4
	Mw (kDa)		3660.1 (3735.3)		4651.8 (4575.8)	
	A260/A280	0.90	0.62	1.03	1.28	1.40
	peak area (%)	3.9	27.2	5.4	56.9	6.6
AAV8oScarlet (3408 bases)	component	LMWS	LMWS	EP	PP	FP
	*s*_w_ (S)	7.8	17.4	45.0	64.2	70.8
	Mw (kDa)			3619.8 (3735.3)		4812.0 (4788.2)
	A260/A280	N.D.^*1^	0.92	0.61	1.10	1.30
	peak area (%)	7.9	4.1	7.3	5.3	75.4
AAV8mCherry-EGFP (4319 bases)	component	EP	PP	PP	PP	FP
	*s*_w_ (S)	45.0	55.2	60.8	65.9	76.7
	Mw (kDa)	3619.8 (3735.3)				5016.5 (5069.6)
	A260/A280	0.67	N.D.^*1^	N.D.^*1^	N.D.^*1^	1.26
	peak area (%)	23.9	3.5^*2^	3.7^*2^	3.8^*2^	65.2

The peaks above the limit of detection (LOD) determined in a previous study were assigned.^10^*s*-values and peak areas are obtained from the *c*(*s*) distributions detected at 230 nm. Mws are calculated using the experimentally determined *s*-value and a previously reported diffusion coefficient.^9^ The values in parentheses are theoretical Mw values determined by the chemical composition. ^*1^ Not determined (peak area cannot be calculated). ^*2^ Peak below the LOD.

### Establishment of a Component Identification Workflow of MW-DGE-AUC

Next, to identify the components other than FP and EP in the DGE-AUC equilibrium profile, we focused on the partial-specific volume (*vbar*), which is the reciprocal of buoyant density and depends on the chemical composition of a particle and the degree of solvation and ion binding to the particle. The *vbar* of a particle composed of proteins and nucleic acids is equal to the weight-averaged *vbar* of the constituent proteins and nucleic acids. The *vbar* values are significantly different for proteins and nucleic acids, typically 0.73 and 0.52 cm^3^/g, respectively. As described, importantly, the *vbar* also varies depending on the composition of the solvent, such as the buffer species and ions, due to solvation and ion binding to the particle. In other words, the *vbar* of AAV particle depends on the solvation state, which is affected by the concentration and type of ions coexisting in the solution, which was shown in previous studies describing the dependence of the *vbar* of proteins and DNA on the concentration of Cs ions.^27−29^

The buoyant density of a particle can be estimated from the position of the band at equilibrium if the relationship between the radial position and the CsCl concentration in a centrifuge tube or cell is obtained. The relationship between the CsCl density and the CsCl concentration is well established (Figure S7). Thus, we attempted to simulate the CsCl concentration gradient at equilibrium using the following equation that expresses the concentration distribution considering the balance between diffusion and sedimentation of Cs ions.^30^
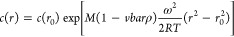
3Here, *r* is the radius (distance from the center of rotation), *r*_0_ is the arbitrary reference radius, *M* is the Mw, *vbar* is the particle partial-specific volume of CsCl, ρ is the solution density, ω is the rotor angular velocity, *R* is the gas constant, and *T* is the absolute temperature. When eq 1 is applied to the DGE-AUC equilibrium profile, only the concentration at the reference radius *c*(*r*_0_) is unknown. Since the total mass of Cs ions in the AUC cell must be conserved during the DGE-AUC experiment, the *isoconcentration point r*_*i*_, a radius where the concentration of Cs ions equals the initial concentration, can be set as *r*_0_. However, it should be noted that the theoretical description of the accurate CsCl concentration gradient is challenging because the high concentration of CsCl generates nonideal conditions and coexisting macromolecules may influence the density gradient.^31^ We next examined whether the simulated density gradient was consistent with the actual density gradient in the presence of AAV particles using CsCl-DGE-UC. The approximate correspondence between the simulated CsCl density gradient without AAV particles and the experimental CsCl density gradient in the presence of AAV particles is shown (Figure S8). These findings were important as they revealed that when the rotor speed, the CsCl concentration in the initial solution, and the meniscus of the solution and of the bottom of the cell were known, the CsCl density gradient at equilibrium could be simulated and was essentially the same for all AAV vectors regardless of the serotype or the length of the encapsidated DNA.

We were then able to calculate the *vbar* of FP, EP, and other components from the band positions in DGE-AUC as follows. As described earlier, the *vbar* of the component determined from the band position at a certain solution density, *vbar*_[Cs]_, is different from the *vbar* in water at 20 °C (i.e., solution density = 0.99820 g/cm^3^), *vbar*_aq_. The *vbar*_aq_ can be calculated from the amino acid and DNA compositions (eq 2) of the macromolecular complex owing to the experimental fact that *vbar* is less influenced by its higher-order structure. We could then establish the relationship between solution density, *vbar*_aq_ or *vbar*_[Cs]_, and DNA length for the EP and FP of AAV2EGFP, AAV5ZsGreen1, AAV8FIXp, AAV8oScarlet, and AAV8mCherry-EGFP. The peaks of EP and FP for these AAV vectors were assigned based on the matching of the A260/A280 value and the peak area between the values from SV-AUC or BS-AUC and DGE-AUC. The average values of *vbar*_[Cs]_ for the two FPs observed in DGE-AUC were used as the values of FPs in AAV2EGFP and AAV5ZsGreen1.

**Figure 5:**
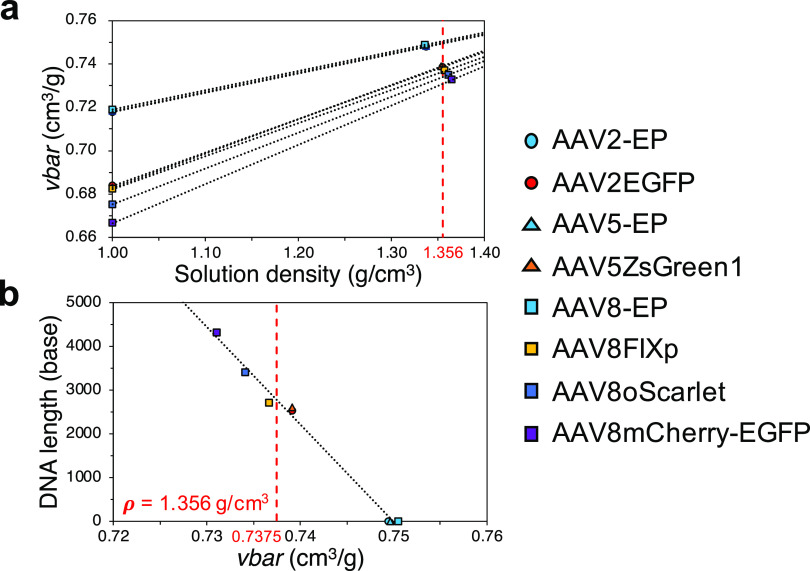
Relationship among solution density, *vbar*, and DNA length. (a) *vbar*_aq_ and *vbar*_[Cs]_ for EP and FP are plotted against solution density. The red dashed line shows the solution density of 1.356 g/cm^3^. (b) Linear correlation between DNA length and *vbar* at a solution density of 1.356 g/cm^3^. The red dashed line shows a *vbar* of 0.7375 cm^3^/g.

**Figure 6:**
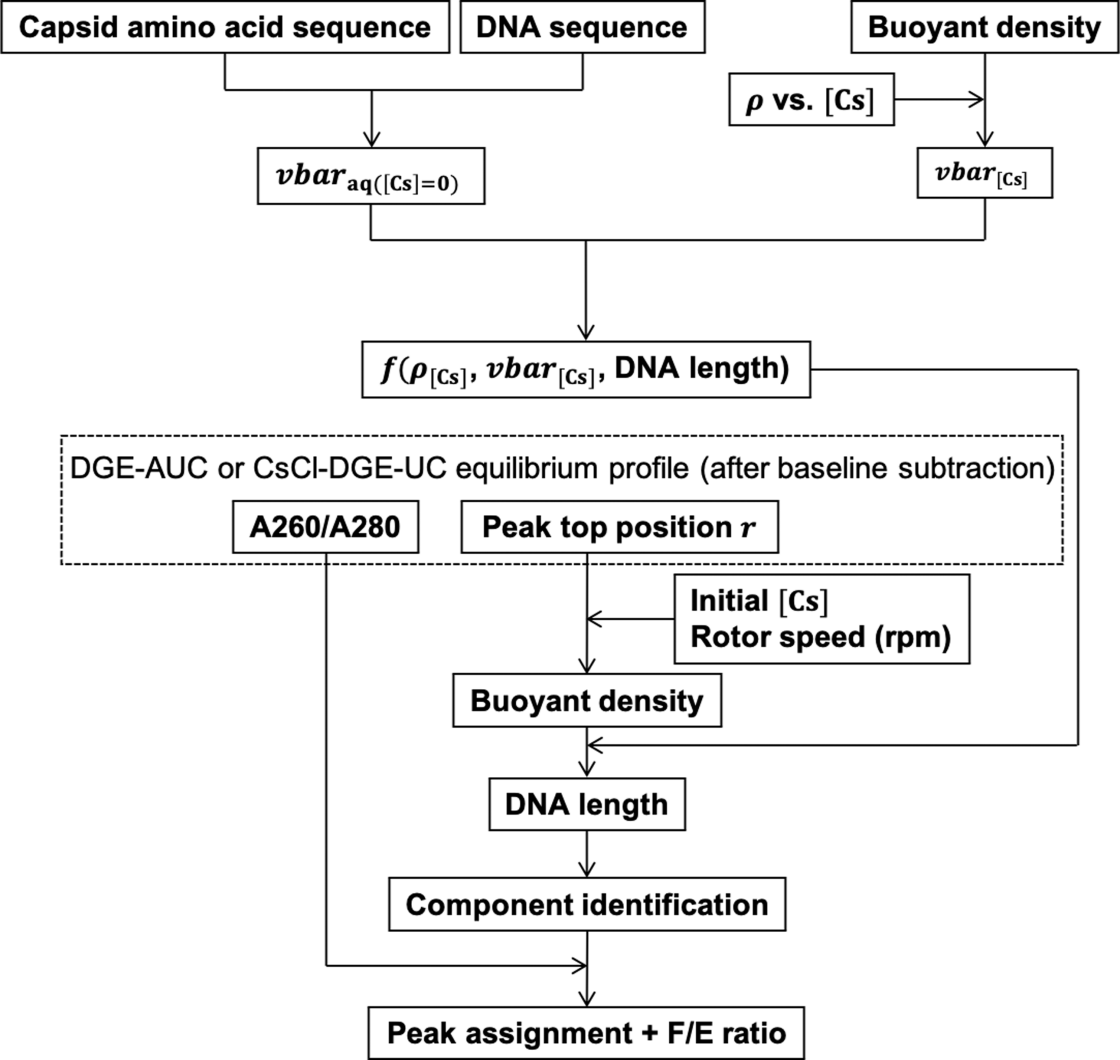
Component identification workflow of MW-DGE-AUC.

Subsequently, we identified the components of the observed peaks in the DGE-AUC equilibrium profile as follows. First, *vbar*_aq_ and *vbar*_[Cs]_ for EP and FP were plotted against solution density (Figure 5a). Assuming a linear relationship between the CsCl concentration and *vbar*_[Cs]_, *vbar*_[Cs]_ for AAVs with various DNA lengths at various solution densities can be calculated. From the plots of calculated *vbar*_[Cs]_ against DNA length, the encapsidated DNA length in the AAV particles could be determined (Figure 5b). For example, peak 4 of AAV8H4C1 (Figure 4a, right) reached 6.80 cm where the solution density was 1.356 g/cm^3^ (CsCl concentration of 2.790 M), meaning that *vbar*_[Cs]=2.790M_ of the component for peak 4 was 0.7375 cm^3^/g. The DNA length of peak 4 was calculated to be 2818 bases from the plots of calculated *vbar*_[Cs]=2.790M_ against DNA length as shown in Figure 5b. Peak 4 was therefore assigned as ExP. The A260 and A280 values could be employed to judge the appropriateness of the peak assignment. In the case of peak 4 of AAV8H4C1, the A260/A280 value of 1.28 supported the proper peak assignment shown above. Figure 6 illustrates the component identification workflow of MW-DGE-AUC.

### Component Identification of AAV8 Vectors by the Developed MW-DGE-AUC Workflow

Here, we applied the component identification workflow of each peak to the characterization of the AAV8 vectors. The obtained DGE-AUC equilibrium profiles and the component identification results are summarized in Figure 4 (right) and Table 2, respectively. These component identification results were compared with those from MW-BS-AUC, as summarized in Table 1.

**Table 2:** Component Identification of AAV8 Vectors by MW-DGE-AUC

AAV8H4C1 (1487 bases)	Peak No.	1	2	3	4	5	6
	DNA length	–32	905	2167	2818	4172	4648
	A260/A280	0.56	0.97	1.10	1.28	1.37	1.49
	peak area (%)	29.8	1.3	43.1	21.0	4.1	0.7
	assignment	EP		FP	ExP	ExP	
AAV8FIXp (2712 bases)	peak no.	1	2	3	4		
	DNA length	266	3020	4275	4719		
	A260/A280	0.61	1.29	1.31	1.31		
	peak area (%)	33.3	52.9	10.2	3.7		
	assignment	EP	FP				
AAV8oScarlet (3408 bases)	peak no.	1	2				
	DNA length	197	3570				
	A260/A280	0.71	1.30				
	peak area (%)	5.6	94.4				
	assignment	EP	FP				
AAV8mCherry-EGFP (4319 bases)	peak no.	1	2	3			
	DNA length	39		4172			
	A260/A280	0.58		1.33			
	peak area (%)	35.5	4.6	59.8			
	assignment	EP	PP	FP			

DNA lengths were calculated by using the relationship among solution density, partial-specific volume, and DNA length. Peak areas were obtained from the DGE-AUC equilibrium profiles detected at 230 nm.

MW-BS-AUC showed that AAV8H4C1 contains two ExPs that encapsidate two or three full-length DNAs or various combinations of DNAs of different lengths, in addition to EP and FP. In MW-DGE-AUC, peaks 1 and 3–5 were identified as EP, FP, and two ExPs, respectively, according to the workflow shown in Figure 6. Considering that PP was not identified from MW-BS-AUC, peak 2 may represent the FP-EP dimer. Peak 6 may be DNA-rich impurities based on the high A260/A280 value. In MW-DGE-AUC, adjacent peaks, such as peaks 3 and 4, are not well-separated. In such a case, Gaussian peak fitting is effective for deriving the population of each component. In fact, the calculated peak area ratio of FP and EP (59.1 and 40.9%, respectively) was in good agreement with that obtained from MW-BS-AUC (56.6 and 43.4%, respectively).

MW-BS-AUC showed that AAV8FIXp contains PP and ExP in addition to EP and FP. From MW-DGE-AUC, peaks 1 and 2 could be identified as EP and FP, respectively, based on the buoyant density and A260/A280 values. The calculated peak area ratio of peaks 2 and 1 (peak 2 61.4%) was slightly inconsistent with that of FP and EP obtained from MW-BS-AUC (FP 67.7%), suggesting that peaks 3 and 4 could correspond to FP and ExP, respectively. PP detected by MW-BS-AUC, which should be located between EP and FP, was not detected as a clear peak, likely because of the low abundance of this component.

MW-BS-AUC revealed that AAV8oScarlet contains PP in addition to EP and FP. However, in MW-DGE-AUC, PP was not detected as a clear peak because of its low abundance, while peaks 1 and 2 were identified as EP and FP, respectively. The calculated peak area ratio of FP and EP (FP 94.4%) was in good agreement with that obtained from MW-BS-AUC (FP 91.2%).

Finally, MW-BS-AUC revealed that AAV8mCherry-EGFP contains three PPs with different DNA lengths, in addition to EP and FP. From MW-DGE-AUC, peaks 1 and 3 were identified as EP and FP, respectively. PP was detected as a broad peak (peak 2) between EP and FP, consistent with the multiple peak appearance of PP in the *c*(*s*) distribution.

In addition, the *vbar*_[*Cs*]_ value for AAV aggregates may be similar to that of EP, PP, or FP, depending on the composition of the aggregates. In such a case, AAV aggregates are located at a similar density to those of EP, PP, or FP. This leads to over- or underestimation of EP or FP because of this phenomenon of overlapping peaks.

### Further Optimization of the DGE-AUC Conditions for Characterizing AAV Vectors

The relationship among solution density, *vbar*, and DNA length enables us to predict the positions in the density gradient of FP, EP, and PP in DGE-AUC, as well as the CsCl-DGE-UC conditions, prior to the measurements. The information on *vbar*_[*Cs*]_ can be utilized for the optimization of the rotor speed and the initial CsCl concentration of the prepared solution for quantitative analysis by DGE-AUC. For example, when AAV6EGFP was examined under the same conditions as other AAV vectors, accurate F/E ratio evaluation was difficult even after prolonged centrifugation because of the poor separation of AAV6-EP located near the meniscus of the solution at 6.12 cm (Figure S10a). In this case, taking the *vbar*_[*Cs*]_ values for EP and FP of AAV6EGFP into account, successful evaluation could be achieved using the optimum conditions for AAV6EGFP analysis (Figure S10b). Clearly, the AAV6-EP peak at ∼6.35 cm was well-separated from the meniscus.

Also, we carried out a DGE-AUC experiment under 60,000 rpm conditions using AAV8EGFP as shown in Figure S11. The observed peaks were getting sharper with increasing rotor speed, while the peaks of EP and two FPs were getting closer. The experimental conditions including rotor speed, rotor type (4-hole rotor and 8-hole rotor), and CsCl concentration should be selected depending on the purpose of the quantitative analysis of each AAV vector.

Furthermore, the optimum conditions can be estimated for the detailed characterization of two FPs by DGE-AUC or appropriate fractionation by CsCl-DGE-UC. Importantly, these approaches for optimization could be used for any AAV serotype of any DNA length.

## Conclusions

In this study, we established a new workflow of DGE-AUC for the characterization of AAV vectors. The correlation of the F/E ratio between DGE-AUC and SV-AUC obtained from measurements using AAV2 showed good agreement. In general, the observed peak areas of the major components in the DGE-AUC equilibrium profile were consistent with those in the *c*(*s*) distribution obtained by BS-AUC; however, in the case where minor components are present, inconsistencies could be observed. These minor components with small absorbance at the detected wavelengths or similar density to major components could be observed as broad peaks or peaks that overlapped with major peaks in the DGE-AUC equilibrium profile, thereby hindering the comprehensive characterization of AAV vectors by DGE-AUC.

Therefore, SV-AUC or BS-AUC should be the first choice method for the accurate and reliable evaluation of the size distribution of AAV vectors for gene therapy. However, DGE-AUC offers great advantages in separating particles with close Mws and *s*-values, which is difficult under the practical conditions of SV-AUC and BS-AUC, such as those of FP1 and FP2. Based on our findings, the cause of the different buoyant densities of FP1 and FP2 is the difference in VP stoichiometry. Thus, taking into account the VP ratio of capsids is expected to improve the accuracy of component identification. Since the density difference may become a CQA in the future, it is important to optimize DGE-AUC because it is a powerful analytical method that can evaluate the particle density heterogeneity. Furthermore, we can analyze the obtained data by performing simple calculations, whereas SV-AUC and BS-AUC require data analysis by model fitting. Thus, SV-AUC or BS-AUC and DGE-AUC are complementary methods for the reliable assessment of the purity of AAV vectors.

## Supporting Information Available

Additional experimental methods of AAV vector production and characterizations, including AUC and CGE measurements; and figures including examination of the DGE-AUC experimental conditions for AAV vector characterizations and calculation of physicochemical properties of AAV particles and CsCl/PBS solution, and the DGE-AUC equilibrium profiles (PDF)
