# Supplementary material for: Applications and Limitations of Equilibrium Density Gradient Analytical Ultracentrifugation for the Quantitative Characterization of Adeno-Associated Virus Vectors
Source: Anal Chem. 2024 Jan 2;96(2):642–51. doi: 10.1021/acs.analchem.3c01955 (PMC10794998; doi:10.1021/acs.analchem.3c01955)
Supplement: Supplementary file 1 — ac3c01955_si_001.pdf [file ac3c01955_si_001.pdf]

Supporting Information

The applications and limitations of equilibrium density gradient analytical ultracentrifugation for the quantitative characterization of adeno-associated virus vector

Kiichi Hirohata[1], Yuki Yamaguchi[1], Takahiro Maruno[1], Risa Shibuya[1], Tetsuo Torisu[1], Takayuki Onishi[1], Hideto Chono[2], Junichi Mineno[2], Yuan Yuzhe[3], Kiyoko Higashiyama[3], Kyoko Masumi-Koizumi[3], Kazuhisa Uchida[3], Takenori Yamamoto[4], Eriko Uchida[4], Takashi Okada[5], Susumu Uchiyama[1*]

[1] Department of Biotechnology, Graduate School of Engineering, Osaka University, 2-1 Yamadaoka, Suita, Osaka, 565-0871, Japan

[2] Takara Bio Inc., 7-4-38 Nojihigashi, Kusatsu, Shiga, 525-0058, Japan

[3] Graduate School of Science, Technology and Innovation, Kobe University, 1-7-49 Minatojima Minamimachi, Chuo-ku, Kobe, 650-0047, Japan

[4] Division of Molecular Target and Gene Therapy Products, National Institute of Health Sciences, 3-25-26 Tonomachi, Kawasaki-ku, Kawasaki-city, Kanagawa, 210-9501, Japan

[5] Institute of Medical Science, The University of Tokyo, 4-6-1, Shirokanedai, Minato-ku, Tokyo, 108-0071, Japan

* Corresponding author: Susumu Uchiyama, Ph.D. E-mail: suchi@bio.eng.osaka-u.ac.jp

Abstract for Supporting Information

We have added Figures with the Methods for the main text as follow. The order of the contents in this information correspond to the order described in the main text.

Materials and Methods

- **AAV8 for FP1 and FP2 preparation and characterizations**
  - Cell culture
  - AAV8 vector preparation
  - Two-cycle CsCl-DGE-UC purification
  - CGE for ssDNA
  - CGE for VP components
- **Root-mean-square-deviation calculation**
- **SV-AUC**
- **BS-AUC**
- **CsCl-DGE-UC for the determination of the formed CsCl density gradient.**

Figures

- **Figure S1. Time required for the CsCl density gradient to reach equilibrium in DGE-AUC.**
- **Figure S2. Examination of the validity of the DGE-AUC equilibrium profile.**
- **Figure S3. Geometry of sector-shape centerpiece (AUC double sector centerpiece with gasket and venting holes).**
- **Figure S4. DGE-AUC experiment of AAV5ZsGreen1.**
- **Figure S5. Illustration of two-cycle CsCl-DGE-UC.**
- **Figure S6. Linear correlation between the *s*-value against encapsidated full-length DNA of the several AAV8 vectors obtained by BS-AUC.**
- **Figure S7. Linear correlation of physicochemical properties of CsCl/PBS solution with 0.001% poloxamer-188.**
- **Figure S8. The relationship between the simulated and experimentally determined density gradient.**
- **Figure S9. DGE-AUC experiments of AAV8 vectors.**
- **Figure S10. Optimization of the DGE-AUC conditions for characterizing AAV vectors.**
- **Figure S11. Comparison the DGE-AUC equilibrium profile under different rotor speed conditions for characterizing AAV vectors.**

References

Materials and Methods

Cell culture

Suspended HEK293T cells were used for AAV8 vector production. Suspended HEK293T cells were maintained with BalanCD HEK293 (FUJIFILM Irvine Scientific, Inc., Santa Ana, CA) with 1% penicillin-streptomycin. Cells were grown as adherent cultures in 5% CO_2 at 37°C.

AAV8 vector preparation

AAV8 vector was generated using the triple plasmid, co-transfection. Briefly, pAAV-Rep&Cap (serotype 8), pAd helper, and transgene (CMV-EGFP) plasmids (Vector Builder, Vector ID: VB010000-9394npt) were co-transfected into suspended HEK293T cells cultured in a bioreactor at a ratio of 1:1:1. AAV8 vector from the transfected cells and the medium was harvested 72 h post-transfection and purified through affinity chromatography using AAVX column (Thermo Fisher Scientific, Waltham, MA) followed by one-cycle CsCl-DGE-UC to separate FP from EP. AAV8 vector purified through affinity chromatography (>2 mL) was dissolved in 2.5 M CsCl / PBS solution with 0.001% poloxamer-188. This final volume of 12 mL solution was loaded into a 13.2 mL ultra-clear ultracentrifuge tube and ultracentrifuged at 34,000 rpm at 20ºC for 72 h using SW 41 Ti rotor and Optima XE-90 (Beckman Coulter). After centrifugation, the band corresponding to FP was collected using Piston Gradient Fractionator (BioComp, Fredericton, Canada) and dialyzed using

Slide-A-Lyzer 10K (Thermo Fisher Scientific).

Two-cycle CsCl-DGE-UC purification

To separate and fractionate low and high buoyant density FPs, two-cycle CsCl-DGE-UC were conducted. The purified AAV8 vector was (>2 mL) dissolved in 2.5 M CsCl / PBS solution with 0.001% poloxamer-188. This final volume of 12 mL solution was loaded into a 13.2 mL ultra-clear ultracentrifuge tube and ultracentrifuged at 34,000 rpm at 20ºC for 72 h using SW 41 Ti rotor and Optima XE-90 (Beckman Coulter). The band corresponding to FP was collected and dissolved in 2.5 M CsCl / PBS solution with 0.001% poloxamer-188. Then, this solution was subjected to another round of ultracentrifugation at 24,000 rpm for 72 h. After centrifugation, the bands corresponding to low and high buoyant density FPs were collected using Piston Gradient Fractionator (BioComp) and dialyzed using Slide-A-Lyzer 10K (Thermo Fisher Scientific).

CGE for ssDNA

10 μL AAV8 solutions (1.0 $\times$ 10^{12} viral genome) were treated with DNAase and ProteaseK to extract the ssDNA from the capsid using the following protocol. To prepare a final volume of 30 μL AAV8 samples, 20 μL of nuclease-free water, 3 μL of 10 $\times$ DNase buffer, 1.5 μL of Benzonase, and 5.5 μL of 1 $\times$ PBS with 0.001% poloxamer-188 were mixed. After this Dnase-treated samples were incubated at 37ºC for 30 min, the samples were transferred to a new tube, to which 10 μL of

500 mM EDTA, 55 μL of 1 × PBS with 0.001% poloxamer-188, and 5 μL of Proteinase K (20 mg/mL) were added to obtain a final volume of 100 μL. Next, the mixture was incubated at 55ºC for 60 min, after which the mixture was heated at 95ºC for 20 min, followed by centrifugation to collect the lysate. Then, the ssDNA was purified according to the protocol mentioned in the QIAquick PCR Purification Kit (QIAGEN, Hilden, Germany) and used as the final collected sample. CGE was measured using a PA800Plus system (Sciex, Framingham, MA). The prepared samples were injected via electrokinetic injection. Detection was performed using a 488-nm laser excitation fluorescence with an emission filter of 520 nm.

CGE for VP components

AAV8 samples for CGE measurement were prepared mostly in accordance with a previously reported procedure.[1] AAV8 solutions with a volume of 10 μL (5.0×10^{10} viral genomes) were denatured and buffer-exchanged following the protocol and the final collected sample was diluted with 50 μL of deionized water for injection. CGE measurement was performed using a PA800Plus system (Sciex). Prepared samples were injected with water plug sample stacking. Detection was performed at 214 nm using a photo diode array detector. To determine the VP stoichiometry, peak areas of the CGE electropherogram detected at 214 nm were divided by the molar extinction coefficient of each VP at 214 nm, reflecting the UV absorbance of peptide bonds and amino acids under denatured conditions.[2,3]

Root-mean-square-deviation calculation

The root-mean-square-deviation (RMSD) of the DGE-AUC profiles every 2 h up to 24 h detected at 230 nm were calculated using the following equation:

$$RMSD = \sqrt{\frac{1}{n}\sum_{i=1}^{n}\{A(t+2)_i - A(t)_i\}^2} \qquad \text{(Equation S1)}$$

where A is the absorbance value at radius (from the center of the rotation) i between the meniscus position of the solution and the bottom of the AUC cell at a certain measurement time t (h).

SV-AUC

AAV2-EP and AAV2EGFP were used, and stocks were diluted to a final absorbance at the 1-cm path length of approximately 0.25 at 280 nm for AAV2-EP and 260 nm for AAV2EGFP. A volume of 390 μ L AAV solution was loaded into the sample sector and a volume of 400 μL of the corresponding solvent was loaded into the reference sector. The sapphire windows and a 12-mm double-sector charcoal-filled epon centerpiece (Beckman Coulter, Brea, CA) were used. Data were collected at 20°C using Optima AUC (Beckman Coulter) at 10,000 rpm with a UV and Rayleigh interference detection system. Data were collected immediately with a radial increment of 10 μm. The sedimentation velocity data were analyzed using the continuous $c(s)$ distribution of the program SEDFIT,[4] where the frictional ratio, meniscus, time-invariant noise, and radial-invariant noise were fitted using a regularization level of 0.68. The sedimentation coefficient (s-value) range of 0–250 S was evaluated with a resolution of 500, and the buffer density and viscosity of PBS were calculated using the program SEDNTERP.[5] Figures of the $c(s)$ distribution were generated using the program GUSSI.[6] The s-value was described as $s_{20,w}$ (s-value in water at 20°C) using the partial-specific volume of AAV2-FP.

BS-AUC

AAV8 vectors were diluted to a final absorbance at the 1-cm path length of 0.25 at 260 nm. Then, 15 μL of the AAV solution and solvent were loaded into a sample or reference reservoir well with a 12-mm band forming centerpiece (Spin Analytical, Berwick, ME) equipped with sapphire windows. A volume of 240 μL or 250 μL PBS / $H_2{}^{18}O$ with 0.001% poloxamer-188 were loaded into the sample or reference sector, respectively. Data were collected at 20°C using Optima AUC (Beckman Coulter) at 20,000 rpm with a UV detection system. Data were collected immediately with a radial increment of 10 μm. The BS-AUC sedimentation data were analyzed using the analytical zone centrifugation $c(s)$ model of the program SEDFIT, where the lamella width, frictional ratio, meniscus, time-invariant noise, and radial-invariant noise were fitted using a regularization level of 0.68. The s-value range of 0–175 S was evaluated with a resolution 350, and the buffer density and viscosity of PBS/$H_2{}^{18}O$ were calculated using the program SEDNTERP. The figures of the $c(s)$ distribution were generated using the program GUSSI. The s-value was described as s_w (s-value as an apparent value under the experimental conditions).

CsCl-DGE-UC for the determination of the formed CsCl density gradient.

The AAV6 vector was dissolved in 2.75 M CsCl / PBS solution with 0.001% poloxamer-188 and loaded into a 12 mL Open-Top polyclear tube (SETON Scientific, Petaluma, CA). The sample was ultracentrifuged for 70 hours at 34,000 rpm at 20ºC using SW 41 Ti rotor and Optima XE-90 (Beckman Coulter). After centrifugation, the sample solution in the tube was continuously fractionated and collected as 0.2-mL fractions using Piston Gradient Fractionator (BioComp). The

density of each fraction was determined from the refractive index measurement using Abbemat 200 (Anton Paar, Graz, Austria).

Figures

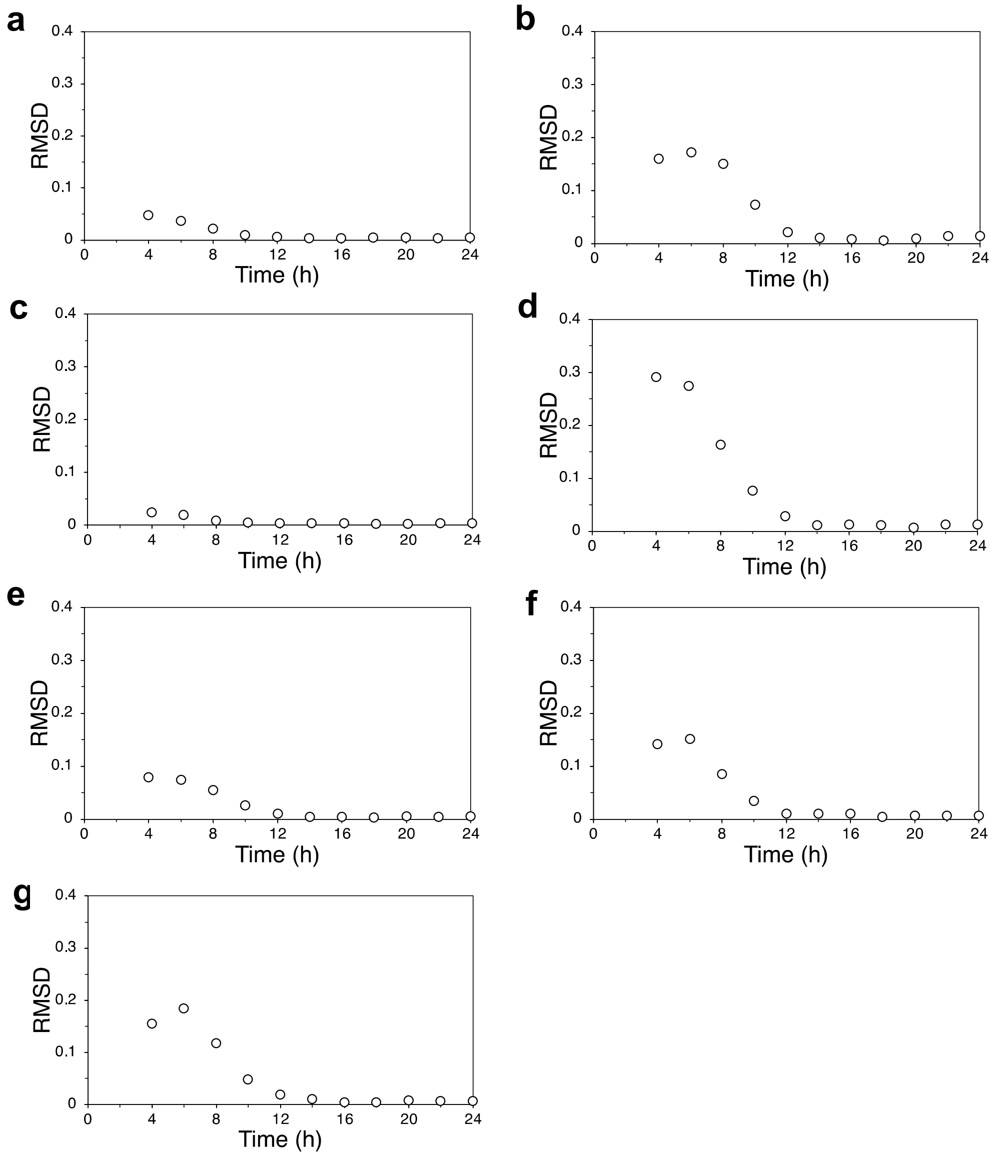

Figure S1. Time required for the CsCl density gradient to reach equilibrium in DGE-AUC. Using Equation S1, the RMSD between the DGE-AUC profiles every 2 h up to 24 h detected at 230 nm were calculated, (a) AAV2EGFP, (b) AAV5ZsGreen1, (c) AAV6EGFP, (d) AAV8H4C1, (e) AAV8EGFP, (f) AAV8oScarlet, and (g) AAV8mCherry-EGFP.

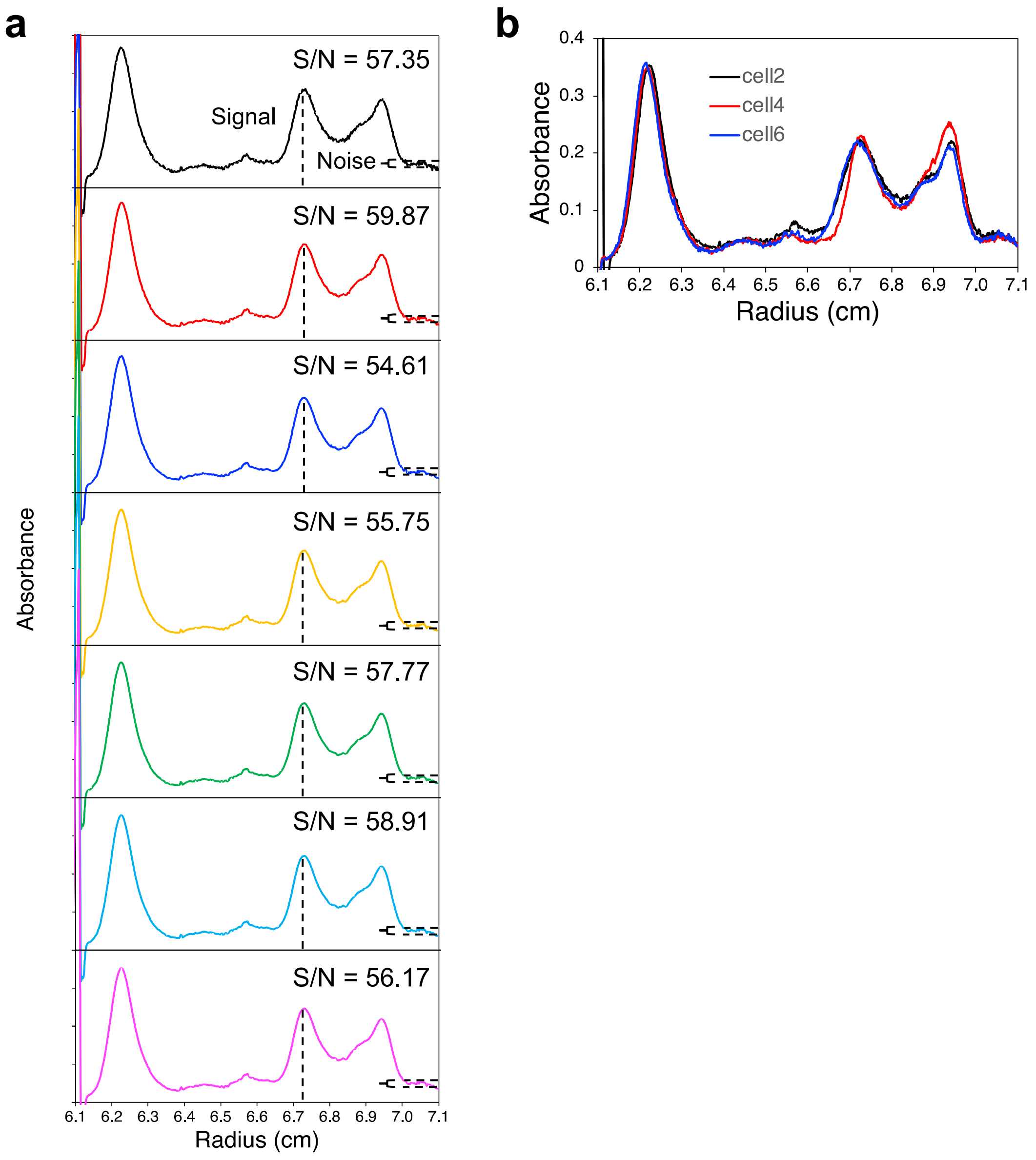

Figure S2. Examination of the validity of the DGE-AUC equilibrium profile. (a) The averaged scan for AAV8EGFP after reaching equilibrium at 42,000 rpm, N=1 (black), N=2 (red), N=4 (blue), N=8 (yellow), N=16 (green), N=32 (sky blue), and N=64 (pink). (b) The overlaid DGE-AUC equilibrium profiles of AAV8EGFP loaded in different cells of the same experiment.

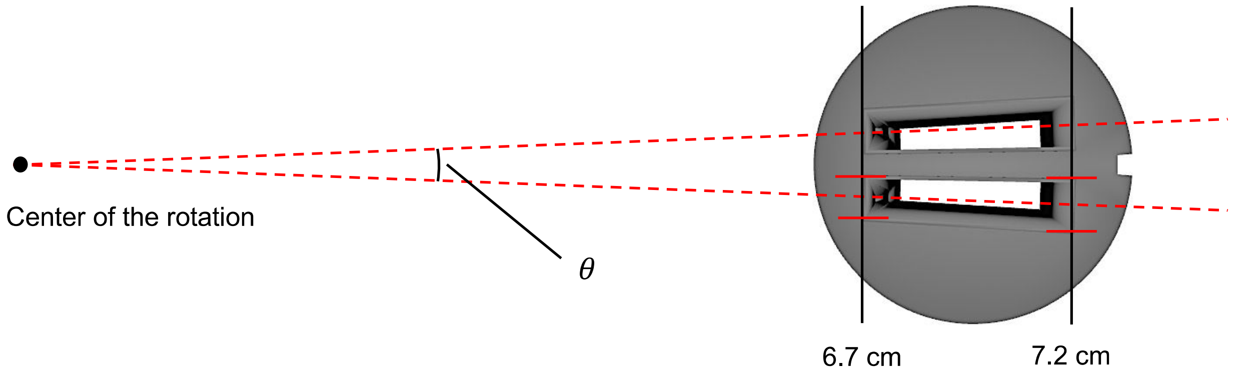

Figure S3. Geometry of sector-shape centerpiece (AUC double sector centerpiece with gasket and venting holes). The width *d* of the sector at *r* from the center of the rotation is calculated by $d = \sqrt{(rcos\theta - r)^2 + (rsin\theta)^2}$. The geometry of sector-shaped centerpiece was obtained from previous study.[7]

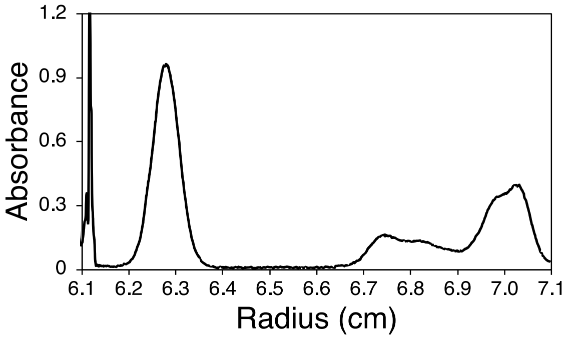

Figure S4. DGE-AUC experiment of AAV5ZsGreen1. The DGE-AUC equilibrium profile for AAV5ZsGreen1 was detected at 230 nm.

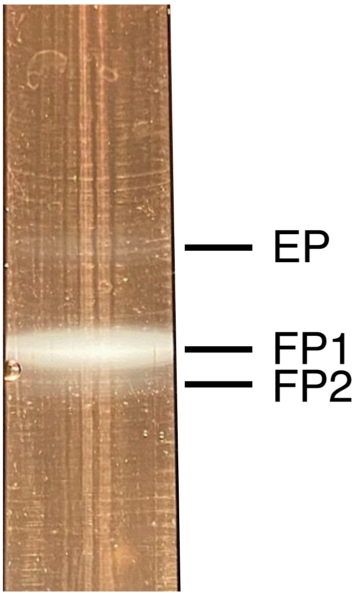

Figure S5. Illustration of two-cycle CsCl-DGE-UC. AAV8EGFP was developed using a suspended HEK293T production system. FP1(low buoyant density) and FP2 (high buoyant density) of AAV2EGFP were separated and fractionated using two-cycle CsCl-DGE-UC after affinity chromatography purification.

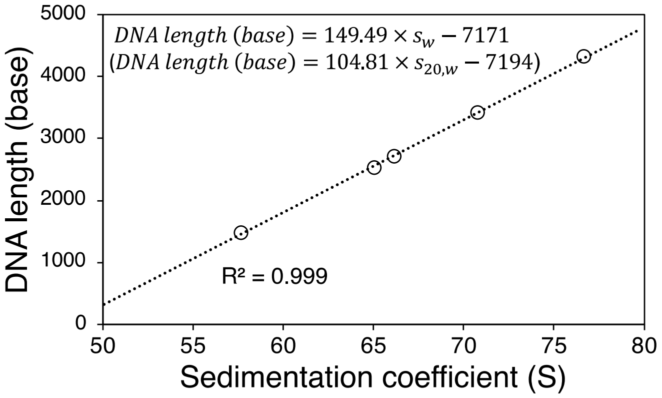

Figure S6. Linear correlation between the *s*-value against encapsidated full-length DNA of the several AAV8 vectors obtained by BS-AUC.

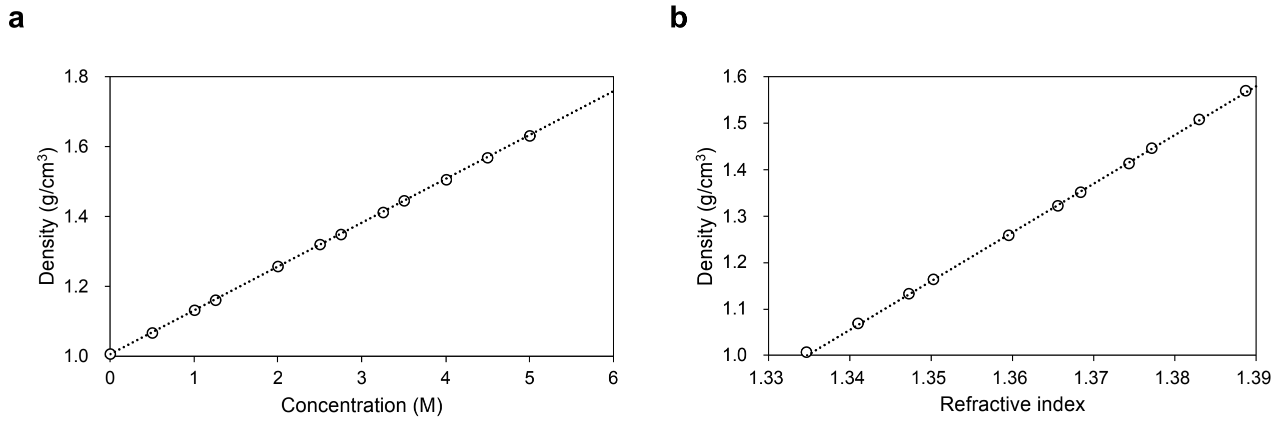

Figure S7. Linear correlation of physicochemical properties of CsCl/PBS solution with 0.001% poloxamer-188. (a) Concentration and density and (b) Refractive index and density.

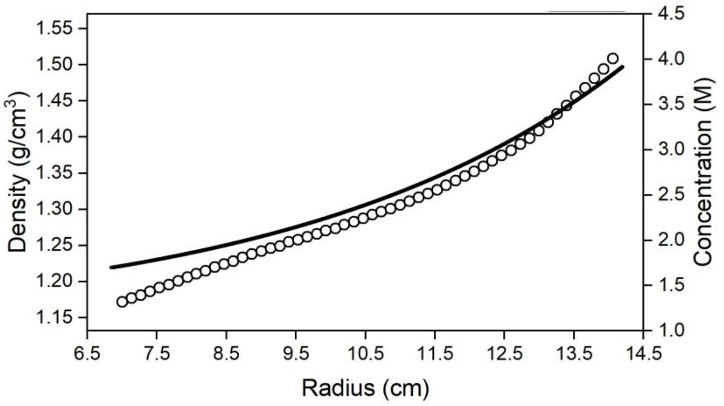

Figure S8. The relationship between the simulated and experimentally determined density gradient. The solid line expresses the simulated CsCl density gradient using Equation 3. The plots show the experimentally determined densities (and concentrations) of each fraction using refractive index measurements after CsCl-DGE-UC.

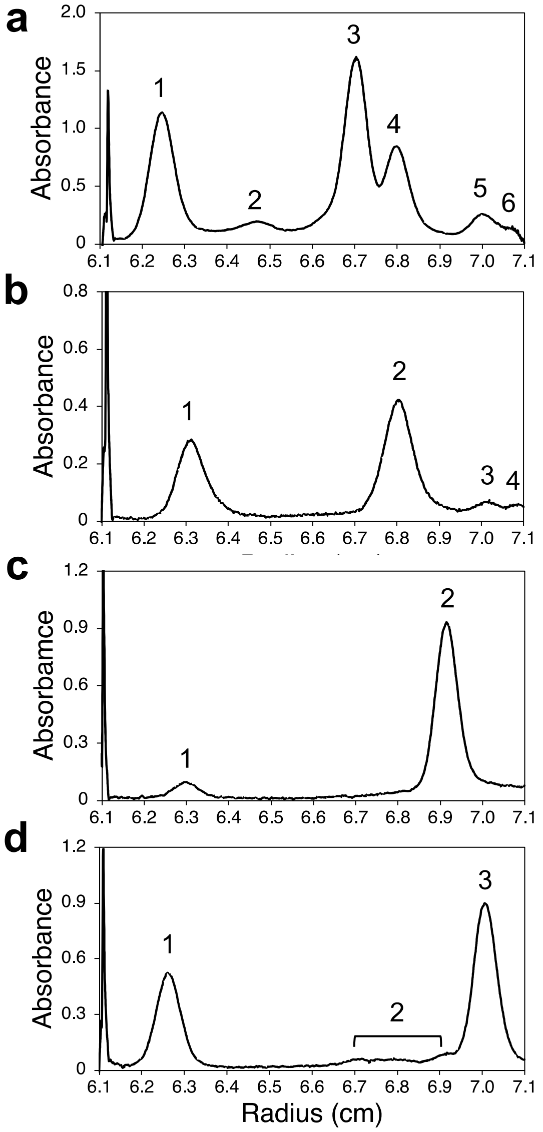

Figure S9. The DGE-AUC experiments of AAV8 vectors. The DGE-AUC equilibrium profiles before baseline subtraction for (a) AAV8H4C1, (b) AAV8FIXp, (c) AAV8oScarlet, and (d) AAV8mCherry-EGFP, respectively. All DGE-AUC equilibrium profiles were detected at 230 nm.

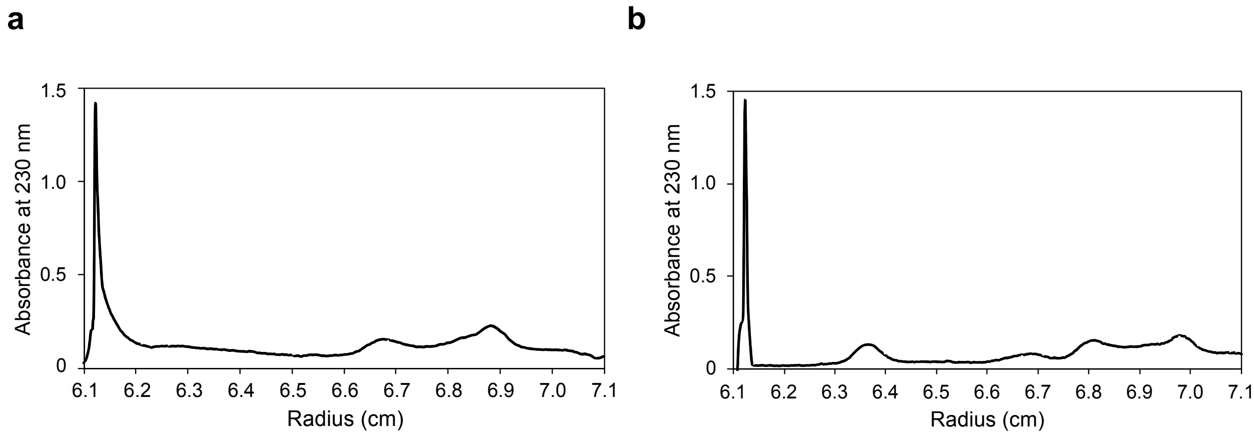

Figure S10. Optimization of the DGE-AUC conditions for characterizing AAV vectors. The DGE-AUC equilibrium profiles of AAV6EGFP detected at 230 nm. (a) CsCl concentration (conc.) is 2.75 M and rotor speed is 42,000 rpm. (b) CsCl conc. is 2.72 M and rotor speed is 45,500 rpm.

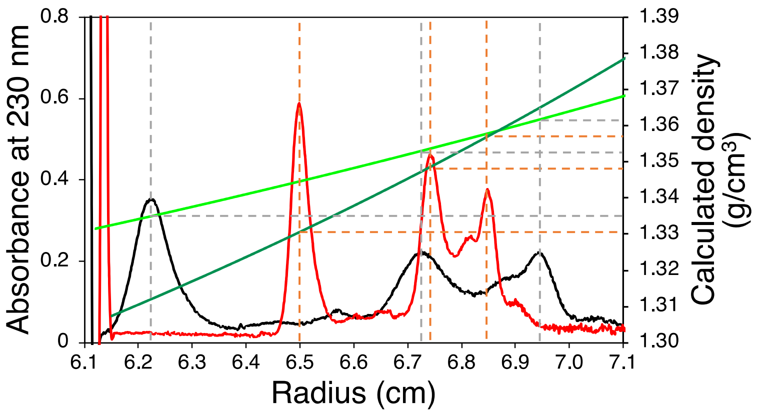

Figure S11. Comparison the DGE-AUC equilibrium profile under different rotor speed conditions for characterizing AAV vectors. The DGE-AUC equilibrium profiles of AAV8EGFP detected at 230 nm. DGE-AUC equilibrium profile of AAV8EGFP under CsCl conc. is 2.75 M and rotor speed is 42,000 rpm (black) and CsCl conc. is 2.70 M and rotor speed is 60,000 rpm (red). Left axis showed the absorbance at 230 nm of the DGE-AUC equilibrium profile and right axis showed the calculated density of CsCl/PBS solution at 42,000 rpm (light green) and 60,000 rpm (dark green) using Equation 3. The gray and orange dashed lines showed the peak top positions and corresponding calculated densities in the DGE-AUC equilibrium profiles at 42,000 rpm and at 60,000 rpm, respectively.

References

1. Kuipers, B.J.H.; Gruppen, H. *J. Agric. Food Chem*. **2007**, 55, 5445–5451.

2. Zhang, C.X.; Meagher, M.M. *Methods Mol. Biol*. **2019**, 1972, 263–270.

3. Oyama, H.; Ishii, K.;Maruno, T.; Torisu, T.; Uchiyama, S. *Hum. Gene Ther*. **2021**, 32, 1403–1416.

4. Schuck, P. *Biophys J.* **1998**, 75(3), 1503-1512.

5. Laue, M.T. *Royal Socie-ty of Chemistry*, **1992**, 90-125.

6. Brautigam, C. A. *Methods Enzymol*. **2015**, 562, 109-133.

7. Desai, A.; Krynitsky, Kuipers, J.; Pohida, T.J.; Zhao, H.; Schuck. P. *PLoS One*. **2016**, 11(8), e0155201.
